# Temporal Trends in Notification and Mortality of Tuberculosis in China, 2004–2019: A Joinpoint and Age–Period–Cohort Analysis

**DOI:** 10.3390/ijerph18115607

**Published:** 2021-05-24

**Authors:** Luqi Wang, Weibing Wang

**Affiliations:** 1 Department of Epidemiology, School of Public Health, Fudan University, Shanghai 200032, China; 20211020147@fudan.edu.cn; 2 Key laboratory of Public Health Safety, School of Public Health, Fudan University, Ministry of Education, Shanghai 200032, China

**Keywords:** tuberculosis, notification, mortality, joinpoint regression model, age–period–cohort model, China

## Abstract

Tuberculosis (TB) remains a major public health problem in China and worldwide. In this article, we used a joinpoint regression model to calculate the average annual percent change (AAPC) of TB notification and mortality in China from 2004 to 2019. We also used an age–period–cohort (APC) model based on the intrinsic estimator (IE) method to simultaneously distinguish the age, period and cohort effects on TB notification and mortality in China. A statistically downward trend was observed in TB notification and mortality over the period, with AAPCs of −4.2% * (−4.9%, −3.4%) and −5.8% (−7.5%, −4.0%), respectively. A bimodal pattern of the age effect was observed, peaking in the young adult (aged 15–34) and elderly (aged 50–84) groups. More specifically, the TB notification risk populations were people aged 20–24 years and 70–74 years; the TB mortality risk population was adults over the age of 60. The period effect suggested that TB notification and mortality risks were nearly stable over the past 15 years. The cohort effect on both TB notification and mortality presented a continuously decreasing trend, and it was no longer a risk factor after 1978. All in all, the age effect should be paid more attention.

## 1. Introduction

Tuberculosis (TB) is one of the major infectious diseases in China, severely threatening the health of the population. Worldwide, TB is also regarded as a major public health problem [1], with about 10 million new cases and 1.5 million deaths reported annually [2]. Since 1949, China has made substantial efforts in decreasing the TB incidence and mortality, but there are still a substantial number of new cases worldwide [3,4]. Therefore, identifying the tuberculosis distribution in the Chinese population and taking relevant public health measures are critical to TB prevention and control in China. In this study, the TB notification and mortality variations from 2004 to 2019 in mainland China were reviewed by a piecewise trend study, demonstrating the effectiveness of current TB public health measures. The TB notification and mortality high-risk groups in the Chinese population were identified by an age–period–cohort (APC) model.

Compared with previous TB incidence and mortality studies in China that focused on the analysis of entire temporal variations, this study paid attention to piecewise trend variations using a joinpoint regression model. It could help to find meaningful turning points or trends in disease incidence and mortality over time. To the best of our knowledge, few studies have described TB incidence and mortality temporal variations in China using this model [3,5,6,7].

This study also aimed to estimate age, period and cohort effects on TB notification and mortality in mainland Chinese residents. Age, period and cohort are usually three key factors influencing disease incidence and mortality [8]. Considering that previous studies in China possibly ignored the interaction between age, period and cohort effects [9,10], we used an age–period–cohort (APC) model to simultaneously calculate these three variables [11]. There were four studies that analyzed TB incidence and mortality using the APC model. Two of them analyzed TB incidence in the United States and Hong Kong [12,13], and the rest analyzed TB mortality in Taiwan and Korea [14,15]. Few such studies were found in China, except for a study that compared only TB incidence among China, India and the United States [16].

## 2. Materials and Methods

### 2.1. Data Source

All data used in this article were extracted from the China Public Health Science Statistics Center (http://www.phsciencedata.cn (accessed on 21 May 2021)). This is a web-based database for selected infectious diseases, collecting data from the infectious disease direct reporting system since 2004. Clinicians were required to complete a standard case report card within a fixed time [3]. The tuberculosis dataset from this website mainly contained the number of notifications and deaths, and the rates of notifications and deaths by age groups and provinces from 2004 to 2017. Additionally, TB notification data for 2018 and 2019 and TB mortality data for 2018 were obtained from the Chinese Center for Disease Control and Prevention. All data were aggregate rather than individual, and we provide the source data in the Supplementary Materials. Subjects who were 85 years of age or older were not included in this study in consideration of analysis requirements. The standard population used in the joinpoint regression analysis was the Chinese Population Census in 2010.

### 2.2. Statistical Analysis

#### 2.2.1. Joinpoint Regression Analysis

Data in this article were analyzed in Joinpoint regression software (version 4.8.0.1, National Cancer Institute, Bethesda, MD, USA), developed by the National Cancer Institute [17]. The grid search method (GSM) was the default modeling method, and the Monte Carlo permutation test was the default optimization method of the model. The Bayesian information criterion (BIC) was used to correct the statistically significant level. The most appropriate joinpoints of the interval piecewise function were finally selected by the above methods [18]. The annual percent change (APC), the average annual percent change (AAPC) and the 95% confidence interval were the main indicators in the joinpoint regression model analysis to describe the variations in temporal trends [19]. There were two terms to describe the temporal variation in the joinpoint regression model: increase (APC > 0, *p* < 0.05); decrease (APC < 0, *p* < 0.05). The results of the statistical tests were two-sided, with values of *p* < 0.05 considered statistically significant [20]. In view of demographic changes, we calculated age-standardized incidence and mortality rates.

#### 2.2.2. Age–Period–Cohort Model Analysis

When the APC model was proposed, it failed to calculate the specific effects of age, period and cohort because there was a colinear relationship between these three variables (i.e., cohort = period–age), which is also called a non-identification problem [21]. It was not until 2000 that Yang Y and Fu successfully distinguished these three effects utilizing an intrinsic estimator (IE) method and demonstrated its feasibility and uniqueness [22,23]. Nowadays, the APC model is regarded as one of the best ways to calculate age, period and cohort effects, with the ability to estimate which factor influenced the expected outcomes, the size of the influence and the variations in the influence [24]. Additionally, it is widely used in epidemiological, demographical and sociological studies [25,26].

The IE method requires 5-year intervals for each age group, and the APC model requires equal intervals for age, period and cohort. Hence, age, period and cohort were divided into 17 groups (0–4, 5–9, ..., 80–84), 3 periods (2004–2008, 2009–2013, 2014–2018) and 19 cohorts (1924–1928, 1929–1933, 1934–1938, ..., 2014–2018), respectively. The number of birth cohorts was calculated by the number of age groups plus the number of periods minus 1. As neighboring birth cohorts partially overlap, the birth cohort is usually described by the middle year of the birth cohort. For patients aged 75–79 years and 80–84 years from 2004 to 2008, their birth cohorts were from 1925 to 1933 and from 1920 to 1928. They are denoted as from 1924 to 1928 and from 1929 to 1933. The APC model is based on a log-linear model and can be written as follows [27]:

Y = log(R) = μ_0_ + α × age_A_ + β × period_P_ + γ × cohort_C_ + ε (1)

where R stands for the expected rates; µ_0_ and ε stand for the intercept item and random error, respectively; α, β and γ are model coefficients and stand for the corresponding age, period and cohort effects, respectively. The relative risk is the exponential value of the coefficient. The analyses of this study were conducted in Stata software (version 15.1, Statacorp, College Station, TX, USA).

## 3. Results

### 3.1. Joinpoint Regression Model Analysis

**Figure 1:**
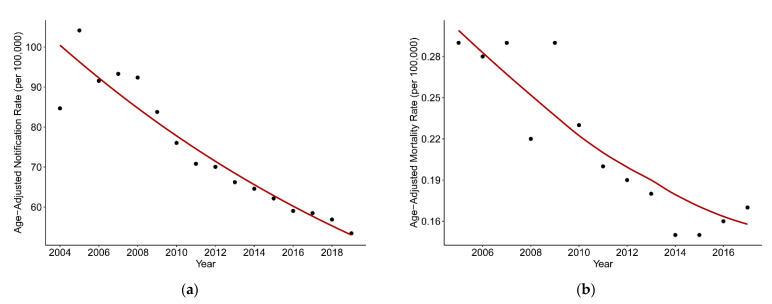
Temporal variations in TB age-standardized rates (per 100,000) in mainland China from 2004 to 2019. (**a**) TB age-standardized notification rate. (**b**) TB age-standardized mortality rate.

Figure 1a shows the temporal variation in TB age-standardized notification and mortality rates since 2004. The TB age-standardized notification rate declined from 84.67 per 100,000 in 2004 to 53.43 per 100,000 in 2019, with an average annual percent change of −4.2% * (−4.9%, −3.4%). Specifically, it increased a little from 2004 to 2007 before obviously decreasing from 2007 to 2019. The highest and the lowest values were observed in 2005 (104.16 per 100,000) and 2019 (53.43 per 100,000), respectively. After excluding two outliers of TB mortality data for 2004 and 2018, the TB age-standardized mortality rate also experienced a decline from 2005 to 2017, with an average annual percent change of −5.8% * (−7.5%, −4.0%). The highest and lowest TB age-standardized mortality rates were 0.3 per 100,000 in 2005 and 0.15 per 100,000 in 2015, respectively. Compared with the TB age-standardized notification rate, the TB age-standardized mortality rate fluctuated more moderately over the period, even showing a rising trend since 2015.

Temporal variations in age-specific TB notification and mortality since 2004 are shown in Table 1. The TB notifications in all age groups generally showed statistically significant downward trends between 2004 and 2019, though the declines in the age groups of 45–49, 55–59 and 65–69 and the increase in the age group of 80–84 were not statistically significant. The greatest reductions in TB notification were observed in the age groups of 0–4 and 5–9, with AAPCs of −14.7% * and −12.3% *, respectively. Age group-stratified TB mortality varied between 2005 and 2017, also showing a significant downward trend in all age groups, with the exception of age groups 15–19 and 50–54. The reductions were also observed in the age groups of 0–4 and 5–9, with AAPCs of −13.6% * and −11.5% *, respectively.

### 3.2. Description Analysis and Age–Period–Cohort Model Analysis

#### 3.2.1. Variation with Age

**Figure 2:**
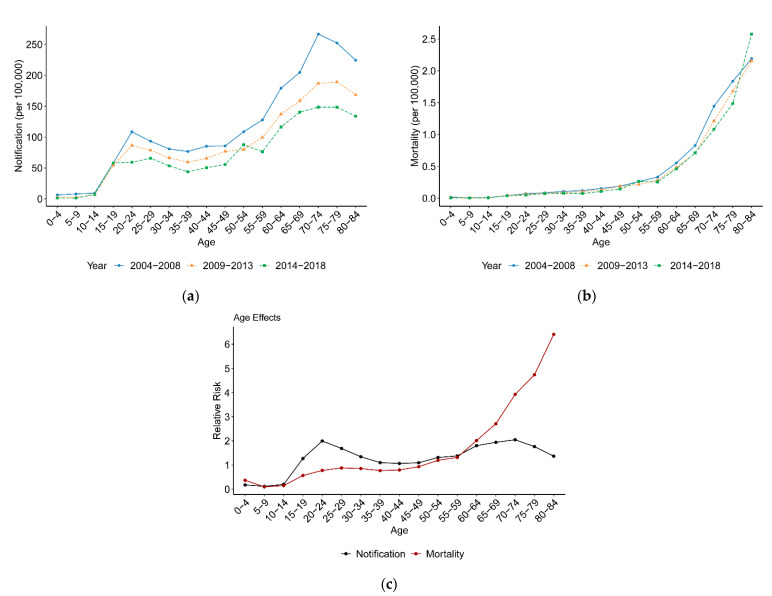
TB notification and mortality variation with age between 2004 and 2018, and the age effects on TB notification and mortality. (**a**) TB notification variation with age. (**b**) TB mortality variation with age. (**c**) Age effects.

Figure 2a,b present TB notification and mortality variations with age between 2004 and 2018. TB age-specific notification maintained stable until the age of 15 and then increased with age. It reached the first peak of notification at the 20–24 age group and then declined to the bottom at the 35–39 age group. Next, it increased again and reached the second peak of notification at the 70–74 age group, in which the value of TB notification was approximately twice that of the first peak. After that, a downward trend with age in TB notification was observed. Additionally, TB mortality maintained stable until the age of 15 and then increased with age, experiencing a slight growth at the 15–59 age group and a sharp growth over the age of 60. Unlike TB notification, no decreasing trend with age was observed in TB mortality.

Actually, for a given period, older people were from earlier birth cohorts; therefore, the increasing TB notification and mortality with age might be due to the age effect or the cohort effect. Figure 2c presents the single age effect on TB notification and mortality risks after controlling for period and cohort effects. At the start, it maintained a low level and started to rise from the age of 15. For TB notification, people aged 15–34 and 50–84 are two risk groups with a relative risk (RR) of > 1, and those aged 20–24 and 70–74 are the highest-risk groups. It should be noted that the RR for the two groups was almost the same at 2.0. This is not consistent with observations from description analysis where TB notification in young adults is twice that in old people. For TB mortality, people over the age of 50 are the risk population (RR > 1), while the RR of TB mortality continuously increased over the age of 60.

#### 3.2.2. Variation with Period

**Figure 3:**
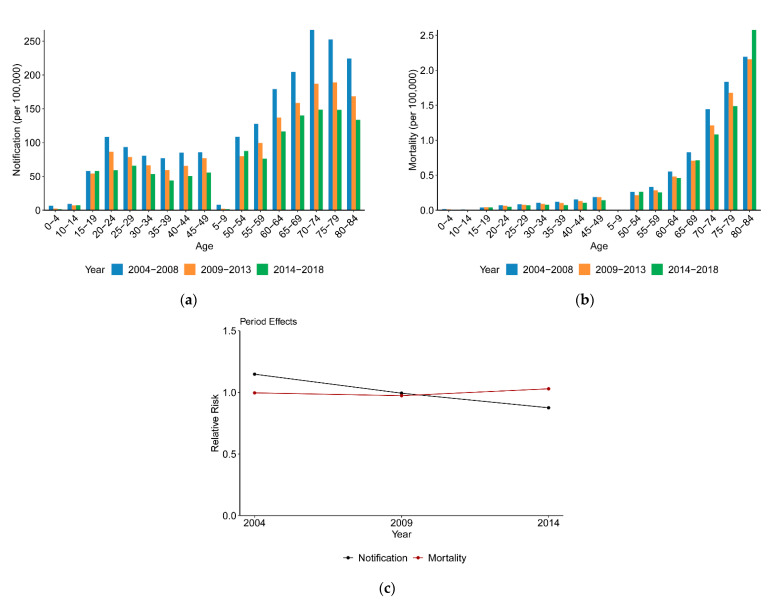
TB notification and mortality variation with period between 2004 and 2018, and the period effects on TB notification and mortality. (**a**) TB notification variation with period. (**b**) TB mortality variation with period. (**c**) Period effects.

Figure 3a shows a downward trend in TB notification of all age groups from 2004 to 2018, which is consistent with the results presented in Table 1. TB mortality showed a minor reduction in young age groups and a moderate reduction in the older age groups from 2014 to 2018 in Figure 3b, except for the 80–84 age group.

Actually, for a given age group, those who survived in recent years also belong to later birth cohorts; thus, the fact that TB incidence and mortality dropped with period might be due to the period effect or the cohort effect. In Figure 3c, after controlling the age and cohort effects, the RR of the period effect on TB notification continuously decreased from 1.15 in the period of 2004–2008 to 0.88 in the period of 2014–2018. For the period effect on TB mortality, the RR maintained stable between 2004 and 2013, followed by a small increase to 1.03 in the period of 2014–2018. From the past to the present, the period effect shifted from a risk factor (RR > 1) to a protective factor (RR < 1) for TB notification, while the RR of the period effect was near 1.0 for TB mortality.

#### 3.2.3. Variation with Cohort

**Figure 4:**
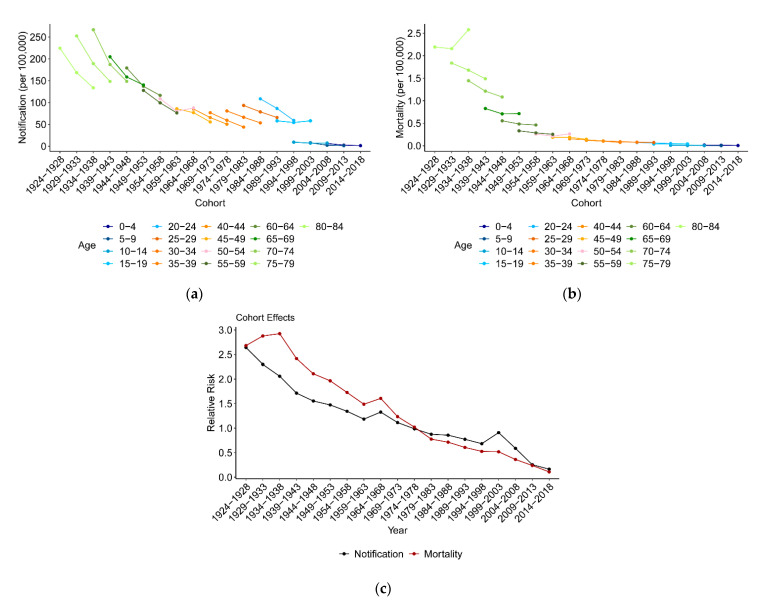
TB notification and mortality variation with birth cohorts between 2004 and 2018, and the cohort effects on TB notification and mortality. (**a**) TB notification variation with cohorts. (**b**) TB mortality variation with cohorts. (**c**) Cohort effects.

Figure 4a,b show TB notification and mortality variations with birth cohorts. TB notification continuously decreased with birth year in all age groups, except for the 50–54 age group born in 1964–1968 and the 15–19 age group born in 1999–2003. TB mortality also decreased with birth cohort, except for the 80–84 age group born in 1934–1938, while this downward trend was less than that of TB notification.

For a given age group, the later cohort were people born in recent years; thus, the decline in notification and mortality in recent cohorts might be due to the cohort effect or the period effect. After controlling the age and period effects, the RR of the cohort effect on TB notification and mortality in general presented a continuously decreasing trend from the earliest birth cohort to the latest. The RR of the period effect on both notification and mortality was 2.6 for the cohort of 1924–1928 and decreased to the lowest value of 0 for the cohort 2014–2018. In fact, the period effect was no longer a risk factor (RR < 1) for TB notification and mortality after birth cohort 1974–1978.

## 4. Discussion

### 4.1. Temporal Variation in Tuberculosis Incidence and Mortality

TB age-standardized notification and mortality rates all showed a significant downward trend over the period, except for the years of 2004–2007. Data in this period fluctuated and dispersed, possibly because the infectious disease network direct reporting system was not perfect at the beginning. This direct reporting system was founded in 2004 [28]. Since 2007, TB notification and mortality started to decline gradually with time, possibly benefitting from a series of interventions and policies introduced by the government. Some studies conducted in other countries have found that TB notification has decreased slowly in recent years [29,30,31], but this was not found in this study.

### 4.2. Age, Period and Cohort Effects

There is a clear pattern in the age effect on TB notification and mortality. The period effect nearly maintained stable over the past 15 years. The cohort effect continuously declined from the earliest cohort (RR = 2.6) to the latest cohort (RR = 0.1). Compared with period and cohort effects, the age effect played a more important role in TB notification and mortality.

The description analysis indicated that TB notification was much higher in the elderly than that in young adults; however, the age–period–cohort model showed that the relative risk of TB notification was actually the same in the elderly and young adults. This reminded us that the results from description analysis might be confounded. The age–period–cohort model could avoid this bias and reveal the true age pattern of disease. Comparing Figure 2a with Figure 2c, the elderly had a much higher TB notification risk due to birth cohort rather than age. People born early were more likely to develop tuberculosis.

The age effect represents the risk differences between different age groups, including changes in physical condition associated with aging and in subjective attitude associated with experience in society. In this study, TB notification and mortality risks were quite low before the age of 15, partially due to the neonatal BCG vaccination program. Then, there was a bimodal distribution with age in the TB notification risk in mainland Chinese residents. One peak was at the 20–24 age group, and the other was at the 70–74 age group. The RRs of these two age groups were nearly the same (RR = 2.0). From a wider perspective, young adults (15–34) and the elderly (50–84) were TB notification risk populations in mainland China. Unlike the bimodal distribution of the TB notification risk, the TB mortality risk increased monotonically with age and sharply increased from the age of 50. The elderly have a high RR of TB mortality mainly because of the definition of TB mortality. It is defined as death during treatment from any cause, which makes TB mortality closely age-dependent [32]. Old people usually suffer from multiple diseases, and they may just be simply dying of old age or other diseases in the process of treatment, causing a high TB mortality risk in the elderly population. Another reason may be the incomplete adjustment of the intrinsic estimator approach, though it allows simultaneous adjustment for age and cohort effects. It has to be reported that China is facing an aging population [33]. The changes in the age distribution of the population are likely to increase the proportion of TB incidence and mortality [34]. By contrast, young adults are less likely to die possibly because tuberculosis is a curable disease as long as it is detected early and rational drug use throughout the course of treatment is followed.

Period effects usually reflect an impact of a given time that directly influences disease incidence and mortality on all age groups or birth cohorts, mainly generated by external macro-factors such as social, economic and medical levels. The decline in TB notification with period suggests the effectiveness of improving economic conditions to reduce tuberculosis progress rates, while this trend was not observed in TB mortality. The RR of the period effect on TB mortality nearly maintained stable from 2004 to 2013 and appeared to rise since 2013, probably induced by the abnormally high TB mortality data for 2018. Over the past 15 years, the RR of the period effect on TB notification and mortality barely changed, which reminded us that more attention should be paid to new detection methods and treatment techniques.

The role of age in variations in disease notification and mortality is often well understood, but the association of birth cohort with disease notification and mortality is difficult to understand. The cohort effect, also called the generation effect, is considered to associate with some specific social events such as wars, the baby boom, the Great Depression and so forth, reflecting different RRs of disease incidence and mortality among different generations [35,36]. In this study, the cohort effect showed that the RR of TB notification and mortality decreased with the birth cohort, and the later people were born, the lower the cohort effect risk became. The values of the cohort effect on TB notification and mortality were less than 1.0 since 1978 when China began to reform and open, and even reached approximately 0.1 in the birth cohort of 2014–2018. A turbulent society, frequent wars, a negative economy and productivity filled in the early years of China, and people born in that time experienced a higher risk of TB notification and mortality. The year of 1978, the beginning of reform and open policy, was a turning point for TB notification and mortality. People born in that time experienced a lower risk of TB notification and mortality. There was a drop in TB notification (Figure 4a) among the cohort born in 1994. The government of China loaned from the World Bank to fully implement the strategy of Directly Observed Treatment Short-course (DOTS) in 13 Chinese provinces. Apparently, this made achievements. It also shows that tuberculosis is a disease closely associated with the economy, society and health services. Compared with age and period effects, the cohort effects of TB notification and mortality are no longer obvious.

This study also has some limitations. For example, because of the data availability and model requirements, the data applied in the age–period–cohort model analysis only contained 15 years. Analysis was not conducted at the gender, province or urban and rural levels; therefore, we could not observe more detailed information that influences TB notification and mortality trends in mainland China. Additionally, the published data of TB notification and mortality were only a fraction of actual TB cases and deaths in the population. The observed TB notification trend reflects the notified TB cases instead of all cases (known and unknown). Similarly, the observed TB mortality trend reflects patients who received treatment instead of all patients. Thus, the true incidence and mortality trends may be different from what has been observed in this study to a certain extent. In conclusion, we hope the analysis of TB notification and mortality trends between 2004 and 2019 and the estimation of the age, period and cohort effects will provide some evidence, references and insights for TB prevention and control in mainland China.

## 5. Conclusions

Tuberculosis notification and mortality maintained a low level at the age of 15. Young adults (15–34) and the elderly (50–84) are two risk groups of tuberculosis notification, especially people aged 20–24 years and 70–74 years. Those aged 60 and over are a risk group of tuberculosis mortality. The period effect helped to decrease tuberculosis notification instead of mortality. Later birth cohorts experienced lower TB notification and mortality risks than earlier birth cohorts, and the value of the cohort effect risk was predicted to become much lower in the future. Moreover, the upward trend of tuberculosis mortality in mainland Chinese residents in recent years deserves our vigilance.

## Figures and Tables

**Table 1:** Age-specific TB incidence and mortality variation with time (per 100,000), 2004–2019.

Age Group (Year)	Notification		Mortality	
	AAPC (%)	95%CI	AAPC (%)	95%CI
0–4	−14.7 *	(−16.5, −12.8)	−13.6 *	(−18.6, −8.4)
5–9	−12.3 *	(−16.6, −7.7)	−11.5 *	(−16.2, −6.6)
10–14	−4.5 *	(−6.1, −2.9)	−5.0 *	(−9.2, −0.6)
15–19	−2.2 *	(−3.6, 0.8)	−0.8	(−3.6, 2.0)
20–24	−4.8 *	(−6.6, 3.0)	−7.2 *	(−9.1, −5.2)
25–29	−1.9 *	(−3.4, −0.3)	−4.1 *	(−6.4, −1.8)
30–34	−4.9 *	(−7.0, −2.7)	−6.0 *	(−8.7, −3.2)
35–39	−5.2 *	(−6.1, −4.3)	−8.1 *	(−10.4, −5.9)
40–44	−3.1 *	(−4.3, −1.9)	−6.7 *	(−8.6, −4.7)
45–49	−0.3	(−1.9, −1.5)	−6.8 *	(−9.2, −4.3)
50–54	−2.0 *	(−3.4, −0.6)	−2.3	(−4.9, 0.3)
55–59	−1.3	(−3.4, 0.8)	−5.8 *	(−7.6, −4.0)
60–64	−4.3 *	(−5.2, −3.4)	−5.5 *	(−8.3, −2.5)
65–69	−1.2	(−2.4, 0.1)	−5.4 *	(−7.7, −3.0)
70–74	−3.4 *	(−4.9, −2.0)	−6.7 *	(−8.6, −4.7)
75–79	−4.9 *	(−6.0, −3.8)	−6.2 *	(−8.3, −4.1)
80–84	2.1	(−1.3, 5.6)	−5.5 *	(−8.2, −2.8)

* indicates that AAPCs are significantly different from zero at the alpha = 0.05 level.

## Data Availability

http://www.phsciencedata.cn (accessed on 21 May 2021).

## Supplementary Materials

The following are available online at https://www.mdpi.com/article/10.3390/ijerph18115607/s1, Excel S1: Data Source.
