# Supplementary material for: Temporal Trends in Notification and Mortality of Tuberculosis in China, 2004–2019: A Joinpoint and Age–Period–Cohort Analysis
Source: Int J Environ Res Public Health. 2021 May 24;18(11):5607. doi: 10.3390/ijerph18115607 (PMC8197385; doi:10.3390/ijerph18115607)
Supplement: Supplementary file 1 [file ijerph-18-05607-s001.zip › revise-track changes+source data/ijerph-1207747-ori-revise-track changes.pdf]

Article

Temporal Trends in Notification and Mortality of Tuberculosis in China, 2004-2019: A Joinpoint and Age-Period-Cohort Analysis

Luqi Wang [1], Weibing Wang [1,2,*]

[1] Department of Epidemiology, School of Public Health, Fudan University, Shanghai 200032, China.
[2] School of Public Health, Fudan University, Key laboratory of Public Health Safety, Ministry of Education, Shanghai, China.
* Correspondence: wwb@fudan.edu.cn

Abstract: Tuberculosis (TB) remains a major public health problem in China and worldwide. In this article, we used a joinpoint regression model to calculate the average annual percent change (AAPC) of TB notification and mortality in China from 2004 to 2019. We also used an age-period-cohort (APC) model based on Intrinsic Estimator (IE) method to simultaneously distinguish the age, period and cohort effects on TB notification and mortality in China. A statistically downward trend was observed in TB notification and mortality over the period, with AAPCs of -4.2%*(-4.9%, -3.4%) and -5.8%(-7.5%, -4.0%), respectively. A bimodal pattern of age effect was observed peaking in the young adults (aged 15-34) and the elderly (aged 50-84) groups. . More specifically, the TB notification risk population were people aged 20-24 and 70-74; TB mortality risk population were adults over the age of 60 . The period effect suggested that TB notification and mortality risk nearly kept stable over the past 15 years. The cohort effect on both TB notification and mortality presented a continuously decreasing trend, and it was no longer a risk factor after 1978. All in all, age effect should be paid more attention.

Keywords: tuberculosis; notification; mortality; joinpoint regression model; age-period-cohort model; China

Citation: Wang, L.; Wang, W. Temporal Trends in Notification and Mortality of Tuberculosis in China, 2004–2019: A Joinpoint and Age–Period–Cohort Analysis. *Int. J. Environ. Res. Public Health* **2021**, *18*, 5607. https://doi.org/10.3390/ijerph18115607

Academic Editor: Paul B. Tchounwou

Received: 18 April 2021
Accepted: 20 May 2021
Published: 24 May 2021

Publisher's Note: MDPI stays neutral with regard to jurisdictional claims in published maps and institutional affiliations.

Copyright: © 2021 by the authors. Licensee MDPI, Basel, Switzerland. This article is an open access article distributed under the terms and conditions of the Creative Commons Attribution (CC BY) license (http://creativecommons.org/licenses/by/4.0/).

1. Introduction

Tuberculosis (TB) is one of the major infectious diseases in China, severely threating the health of people. Worldwide, TB is also regarded as a major public health problem [1], with about 10 million new cases and 1.5 million deaths reported annually [2]. Since 1949, China has made substantial efforts in decreasing the TB incidence and mortality, but there are still substantial number of new cases worldwide [3,4]. Therefore, identifying tuberculosis distribution in Chinese population and taking relevant public health measures are critical to TB prevention and control in China. In this study, the TB notification and mortality variation from 2004 to 2019 in China mainland were reviewed by piecewise trend study, demonstrating the effectiveness of current TB public health measures. The TB notification and mortality high-risk groups in Chinese population were identified by an age-period-cohort (APC) model.

Compared with previous TB incidence and mortality studies in China that focused on the analysis of entire temporal variation, this study paid attention to piecewise trend variation using a joinpoint regression model. It could help to find meaningful turning points or trends in disease incidence and mortality over time. To the best of our knowledge, few studies described TB incidence and mortality temporal variation in China using this model [3,5-7].

This study also aimed to estimate age, period and cohort effects on TB notification and mortality in China mainland residents. Age, period and cohort usually are three key factors influencing disease incidence and mortality [8]. Considering previous studies in

China possibly ignored the interaction between age, period and cohort effects [9,10], we used an age-period-cohort (APC) model to simultaneously calculate these three variables [11]. There were four studies that analysed TB incidence and mortality using the APC model. Two of them analysed TB incidence in the United States and Hong Kong [12,13], and the rest analysed TB mortality in Taiwan and Korea [14,15]. Few such studies were found in China, except for a study that compared only TB incidence among China, India and the United States [16].

2. Materials and Methods

2.1. Data Source

All data used in this article were extracted from China Public Health Science Statistics Centre (http://www.phsciencedata.cn). This is a web-based database for selected infectious disease, collecting data from infectious diseases direct reporting system since 2004. Clinicians were required to complete a standard case report card within a fixed time [3]. The tuberculosis dataset from this website mainly contained the number of notification and death, and the rates of notification and death by age groups and provinces from 2004 to 2017. . Additionally, TB notification data for 2018 and 2019, and TB mortality data for 2018 were obtained from Chinese Center for Disease Control and Prevention. All data were aggregate rather than individual, and we provided the source data in Supplement Table. Subjects who were 85 years of age or older were not included in this study in consideration of analysis requirements. Standard population used in joinpoint regression analysis was the Chinese Population Census in 2010.

2.2. Statistical Analysis

2.2.1. Joinpoint Regression Analysis

Data in this article were analysed in Joinpoint regression software (version 4.8.0.1), developed by National Cancer Institute [17]. Grid search method (GSM) was the default modeling method and Monte Carlo permutation test was the default optimization method of model. Bayesian information criterion (BIC) was used to correct the statistically significant level. The most appropriate joinpoints of interval piecewise function was finally selected by above methods [18]. The annual percent change (APC), the average annual percent change (AAPC) and 95% confidence interval were main indicators in joinpoint regression model analysis to describe the variations in temporal trend [19]. There were two terms to describe the temporal variation in joinpoint regression model: increase (APC>0, P<0.05), decrease (APC<0, P<0.05). The results of the statistical tests were two-sided, with values of $P < 0.05$ considered statistically significant [20]. In view of demographic changes, we calculated age-standardized incidence and mortality rates.

2.2.2. Age-Period-Cohort Model Analysis

When the APC model was proposed, it failed to calculate the specific effects of age, period and cohort because there was a colinear relationship between these three variables (*i.e.* cohort=period-age), which was also called a non-identification problem [21]. It was not until in 2000that Yang Y and Fu successfully distinguished these three effects utilizing an intrinsic estimator (IE) method and demonstrated its feasibility and uniqueness [22,23]. Nowadays, the APC model is regarded as one of the best ways to calculate the age, period and cohort effects, with the ability to estimate that which factor influenced the expected outcomes, the size of the influence and the variations of the influence [24]. And it is widely used in epidemiological, demographical and sociological studies [25,26].

The IE method requires 5-year of each age group and the APC model requires the equal intervals of age, period and cohort. Hence, age, period and cohort were divided into 17 groups (0-4, 5-9, ..., 80-84), 3 periods (2004-2008, 2009-2013, 2014-2018) and 19 cohorts (1924-1928, 1929-1933, 1934-1938, ..., 2014-2018), respectively. The number of birth cohort was calculated by number of age groups plus number of periods minus 1. Because neighboring birth cohorts partially overlap, it is usually described by the middle year of the

birth cohort. For patients aged 75-79 years and 80-84 years from 2004 to 2008, their birth cohorts were from 1925 to 1933 and from 1920 to 1928. They are denoted as from 1924 to 1928 and from 1929 to 1933. The APC model is based on a log-linear model and can be written as following [27]:

$$Y=\log(R)=\mu_0+\alpha\times age_A+\beta\times period_P+\gamma\times cohort_C+\varepsilon \quad (1)$$

R stands for the expected rates. μ_0 and ε stand for the intercept item and random error, respectively. α, β, and γ are model coefficients and stand for the corresponding age, period and cohort effects, respectively. The relative risk is exponential value of the coefficient. The analyses of this study were conducted in Stata software (version 15.1).

3. Results

3.1. Joinpoint Regression Model Analysis

Fig.1a showed that the temporal variation in TB age-standardized notification and mortality rates since 2004. The TB age-standardized notification rate declined from 84.67 per 100,000 in 2004 to 53.43 per 100,000 in 2019, with an average annual percent change of -4.2%*(-4.9%, -3.4%). Specifically, it increased a little in the from 2004 to2007 before obviously decreasing from 2007 to2019. The highest and the lowest values were observed in 2005 (104.16 per 100,000) and 2019 (53.43 per 100,000), respectively. After excluding two outliers of TB mortality data for 2004 and 2018, the TB age-standardized mortality rate also experienced a decline from 2005 to 2017, with an average annual percent change of -5.8%*(-7.5%, -4.0%). The highest and lowest TB age-standardized mortality rates were 0.3 per 100,000 in 2005 and 0.15 per 100,000 in 2015, respectively. Compared with TB age-standardized notification rate, the TB age-standardized mortality rate fluctuated more moderately over the period, even showed a rising trend since 2015.

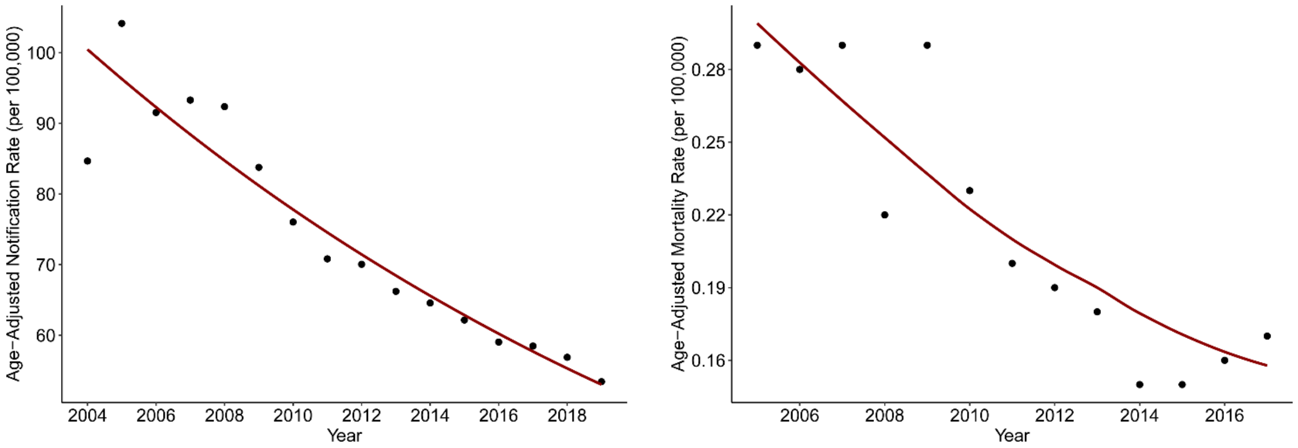

(**a**) (**b**)

Figure 1. Temporal variations in TB age-standardized rates (per 100,000) in China mainland from 2004 to 2019. (a) TB age-standardized notification rate. (b) TB age-standardized mortality rate.

Temporal variation in age-specific TB notification and mortality since 2004 were shown in table 1. The TB notifications in all age groups generally showed statistically significant downward trends between 2004 and 2019, though the declines in the age groups of 45-49, 55-59, 65-69 and the increase in the age group of 80-84 were not statistically significant. The greatest reductions in TB notification were observed in the age groups of 0-4 and 5-9 with AAPCs of -14.7%* and -12.3%*, respectively. Age group stratified TB mortality varied between 2005 and 2017, also showing a significant downward trend in all age groups, with the exception of age groups 15-19 and 50-54. The reductions were also observed in the age groups of 0-4 and 5-9 with AAPCs of -13.6%* and -11.5%*, respectively.

Table 1. Age-specific TB incidence and mortality variation with time (per 100,000), 2004-2019.

Age group (year)	Notification		Mortality	
	AAPC(%)	95%CI	AAPC(%)	95%CI
0-4	-14.7*	(-16.5, -12.8)	-13.6*	(-18.6, -8.4)
5-9	-12.3*	(-16.6, -7.7)	-11.5*	(-16.2, -6.6)
10-14	-4.5*	(-6.1, -2.9)	-5.0*	(-9.2, -0.6)
15-19	-2.2*	(-3.6, 0.8)	-0.8	(-3.6, 2.0)
20-24	-4.8*	(-6.6, 3.0)	-7.2*	(-9.1, -5.2)
25-29	-1.9*	(-3.4, -0.3)	-4.1*	(-6.4, -1.8)
30-34	-4.9*	(-7.0, -2.7)	-6.0*	(-8.7, -3.2)
35-39	-5.2*	(-6.1, -4.3)	-8.1*	(-10.4, -5.9)
40-44	-3.1*	(-4.3, -1.9)	-6.7*	(-8.6, -4.7)
45-49	-0.3	(-1.9, -1.5)	-6.8*	(-9.2, -4.3)
50-54	-2.0*	(-3.4, -0.6)	-2.3	(-4.9, 0.3)
55-59	-1.3	(-3.4, 0.8)	-5.8*	(-7.6, -4.0)
60-64	-4.3*	(-5.2, -3.4)	-5.5*	(-8.3, -2.5)
65-69	-1.2	(-2.4, 0.1)	-5.4*	(-7.7, -3.0)
70-74	-3.4*	(-4.9, -2.0)	-6.7*	(-8.6, -4.7)
75-79	-4.9*	(-6.0, -3.8)	-6.2*	(-8.3, -4.1)
80-84	2.1	(-1.3, 5.6)	-5.5*	(-8.2, -2.8)

* indicates that AAPC are significantly different from zero at the alpha=0.05 level

3.2. Description Analysis and Age-Period-Cohort Model Analysis

3.2.1. Variation with Age

Fig.2a and 2b present TB notification and mortality variations with age between 2004 and 2018. TB age-specific notification maintained stable until the age of 15 and then increased with age. It reached the first peak of incidenceat the 20-24 age group and then declined to the bottom at the 35-39 age group. Next, it increased again and reached the second peak of incidence at the 70-74 age group, in which the value of TB incidence was approximately twice that of the first peak. After that a downward trend with age in TB notification was observed. Also, TB mortality kept stable until the age of 15 and then increased with age, experiencing a slight growth at the 15-59 age groupand a sharp growth over the age of 60. Unlike TB notification, no decreasing trend with age was observed in TB mortality.

Actually, for a given period, older people were from earlier birth cohorts, so therefore the increasing TB notification and mortality with age might be due to age effect or cohort effect. Fig.2c presents the single age effect on TB notification and mortality risk after controlling for period and cohort effects. At start, it maintained a low level and started to rise from the age of 15. For TB notification, people who aged 15-34 and 50-84 are two risk groups with a relative risk (RR) >1, and those aged 20-24 and 70-74 are the highest risk groups. It should be noticed that the RR for the two groups was almost the same of 2.0.It is not consistent with observation from description analysis where TB notification in young adults is twice that of in old people. For TB mortality, people over the age of 50 are the risk population (RR>1), while the RR of TB mortality continuously increased over the age of 60.

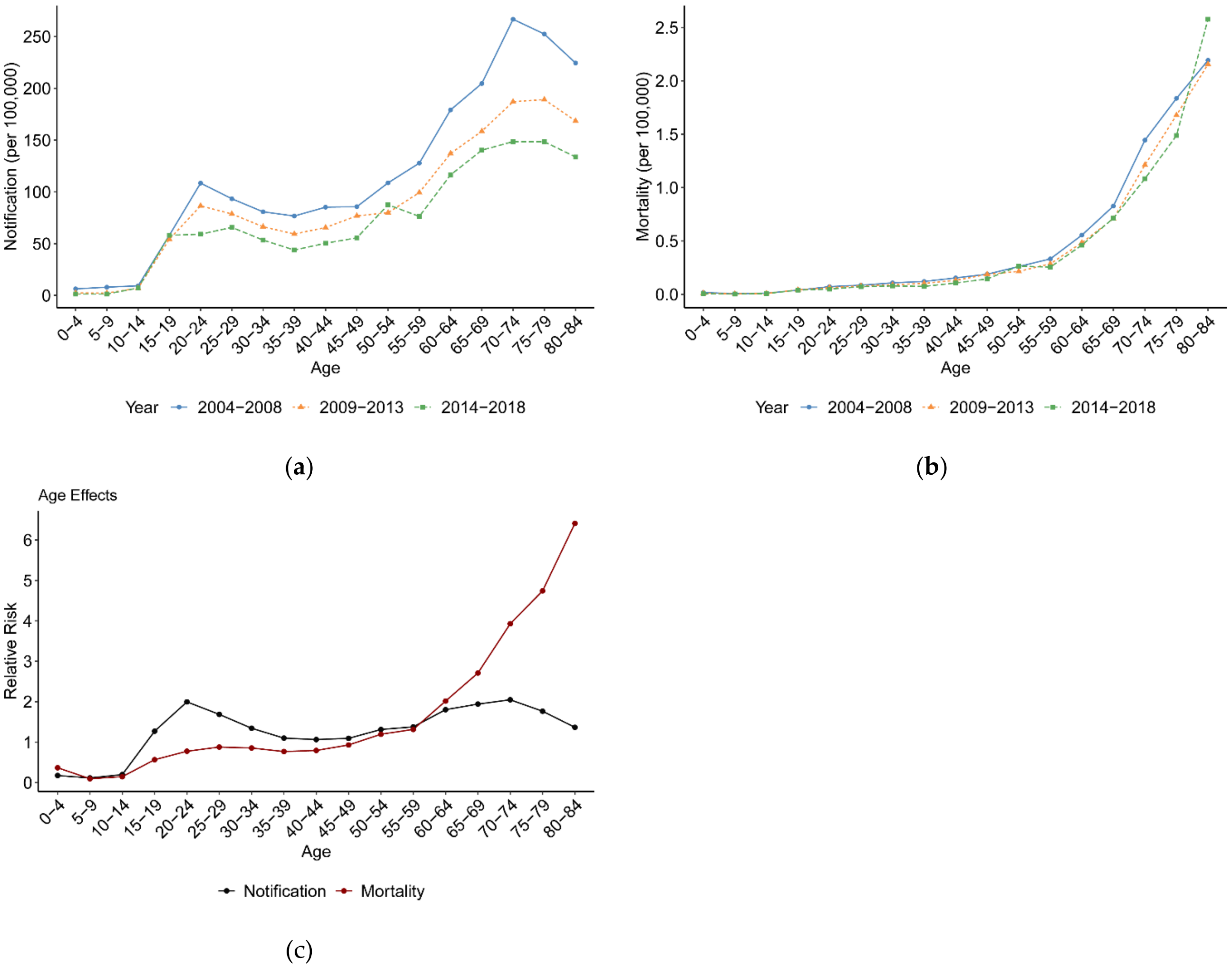

Figure 2. TB notification and mortality variation with age between 2004 and 2018, and the age effects on TB notification and mortality. (a) TB notification variation with age. (b)TB mortality variation with age. (c) age effects.

3.2.2. Variation with Period

Fig.3a shows a downward trend in TB notification of all age groups from 2004 to 2018, which is consistent with the results presented in table 1. TB mortality showed a minor reduction in young age groups and a moderate reduction in the older age groups from 2014 to 2018 in Fig.3b, except for the 80-84 age group.

Actually, for a given age group, those who survived in recent years also belong to later birth cohorts, thus the fact that TB incidence and mortality dropped with period might due to period effect or cohort effect. In Fig.3c, after controlling the age and cohort effects, the RR of the period effect on TB notification continuously decreased from 1.15 in the period of 2004-2008 to 0.88 in the period of 2014-2018. For the period effect of TB mortality, the RR kept stable between 2004 and 2013, followed by a small increase to 1.03 in period 2014-2018. From the past to the present now, the period effectshifted from a risk factor (RR>1) to a protective factor (RR<1) for TB notification, while the RR of period effect was near 1.0 for TB mortality.

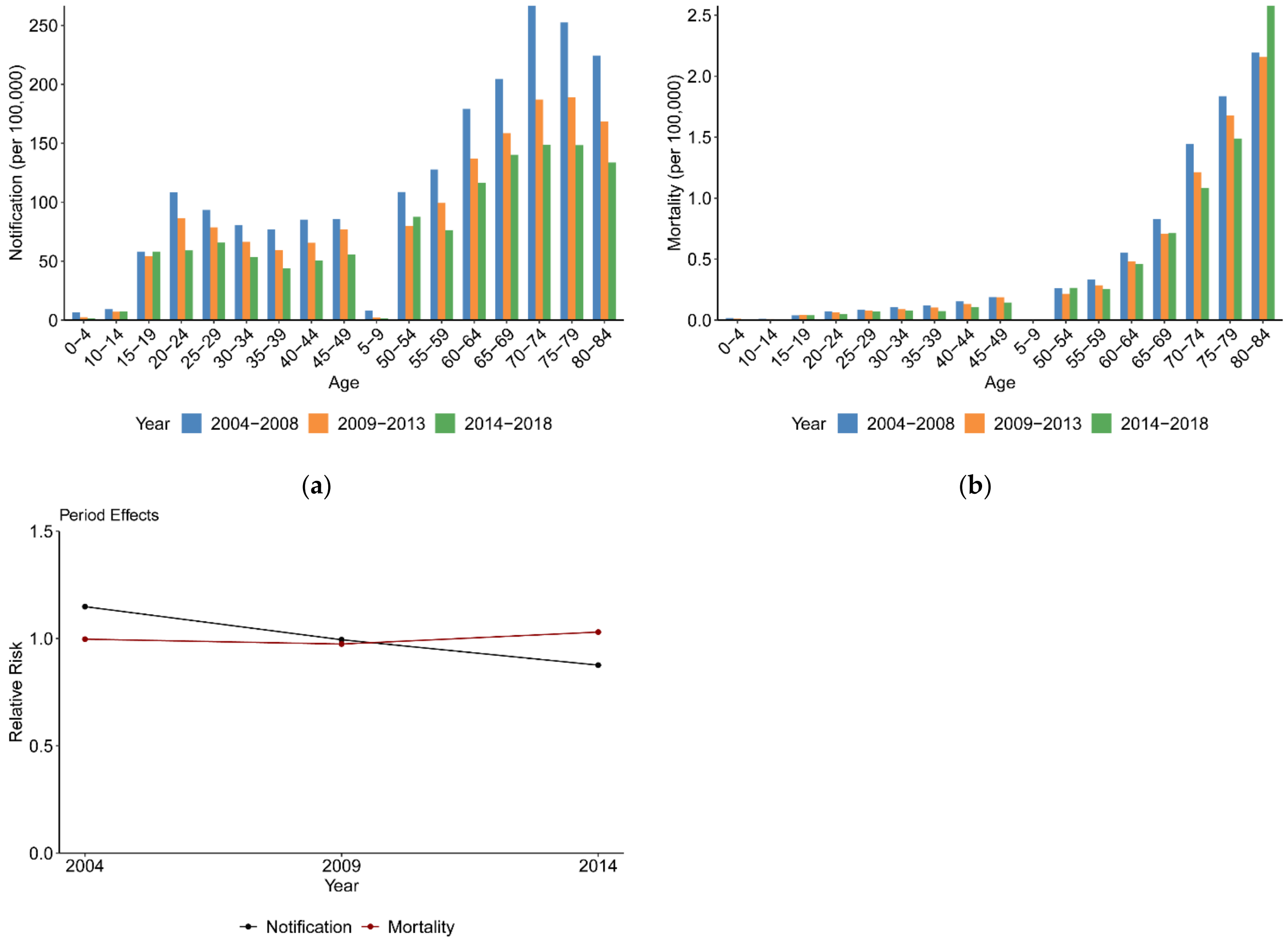

(**a**) (**b**) (c)

Figure 3. TB notification and mortality variation with period between 2004 and 2018, and the period effects on TB notification and mortality. (a) TB notification variation with period. (b)TB mortality variation with period. (c) period effects.

3.2.3. Variation with Cohort

Fig. 4a and 4b show TB notification and mortality variations with birth cohorts. TB notification continuously decreased with birth year in all age groups, except for the 50-54 age group born in 1964-1968 and the 15-19 age group born in 1999-2003. TB mortality also decreased with birth cohort, except for the 80-84 age group born in 1934-1938, while this downward trend was less than that of TB notification.

For a given age group, the later cohort were people born in recent years, thus the declined notification and mortality in recent cohort might be due to cohort effect or period effect. After controlling the age and period effects, the RR of the cohort effect on TB notification and mortality in general presented a continuously decreasing trend from the earliest birth cohort to the latest. The RR of period effect of both notification and mortality was 2.6 for the cohort of 1924-1928 and decreased to the lowest value of 0for the cohort 2014-2018. In fact, the period effect was no longer a risk factor (RR<1) for TB notification and mortality after birth cohorts 1974-1978.

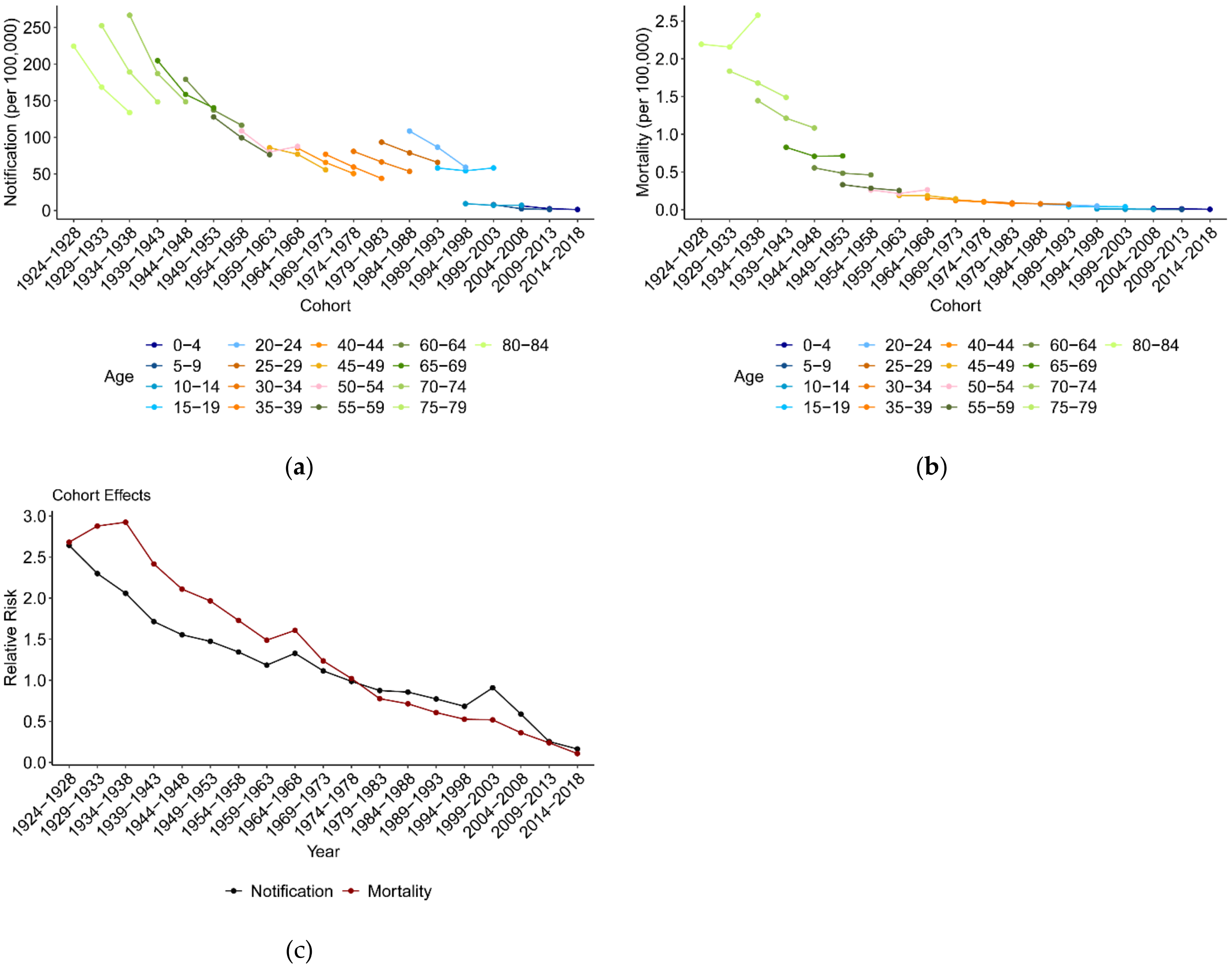

Figure 4. TB notification and mortality variation with birth cohorts between 2004 and 2018, and the cohort effects on TB notification and mortality. (a) TB notification variation with cohorts. (b)TB mortality variation with cohorts. (c) cohort effects.

4. Discussion

4.1. Temporal Variation in Tuberculosis Incidence and Mortality

TB age-standardized notification and mortality rates all showed a significant downward trend over the period, except for the years of 2004-2007. Data in this period fluctuated and dispersed, possibly because the infectious disease network direct reporting system was not perfect at the beginning. This direct reporting system was founded in 2004[28]. Since 2007, TB notification and mortality started to decline gradually with time, possibly benefited from a series of interventions and policies introduced by the government. Some studies conducted in other countries found that TB notification have decreased slowly in recent years [29-31], but it has not been found in this study.

4.2. Age, Period and Cohort Effects

There is a clearly pattern in age effect on TB notification and mortality. The period effect nearly kept stable over the past 15 years. The cohort effect continuously declined from the earliest cohort (RR=2.6) to the latest cohort (RR=0.1). Compared with period and cohort effects, the age effect played a more important role in TB notification and mortality.

The description analysis indicated that TB notification was much higher in the elderly than that in young adults; however, the age-period-cohort model showed that the relative risk of TB notification was actually the same in the elderly and young adults. This reminded us that the results from description analysis might be confounded. The age-

period-cohort model could avoid this bias and reveal the true age pattern of disease. Comparing Fig.2a with Fig.2c, the elderly had a much higher TB notification risk due to birth cohort rather than age. People born early were more likely to develop tuberculosis.

The age effect represents the risk differences between different age groups, including changes in physical condition associated with aging and in subjective attitude associated with experience in society. In this study, TB notification and mortality risk were quite low before the age of 15 partially due to the neonatal BCG vaccination program. Then there is a bimodal distribution with age in TB notification risk in China mainland residents. One peak was at the 20-24 age group, and the other was at 70-74 age group. The RR of these two age groups were nearly the same (RR=2.0). From a wider perspective, young adults (15-34) and the elderly (50-84) were TB notification risk population in China mainland. Unlike the bimodal distribution of TB notification risk, the TB mortality risk increased monotonically with age and sharply increased from the age of 50. The elderly have a high RR of TB mortality mainly because of the definition of TB mortality. It is defined as the death during treatment from any cause, which makes the TB mortality closely age-dependent [32]. Old people usually suffer from multiple diseases and they may just be simply dying of old age or other diseases in the process of treatment, causing high TB mortality risk in the elderly population. Another reason may be the incomplete adjustment of the intrinsic estimator approach, though it allows simultaneous adjustment for age and cohort effects. It has to be reported that China is facing an aging population [33]. The changes in age distribution of population are likely to increase the proportion of TB incidence and mortality [34]. By contrast, young adults are less likely to die possibly because tuberculosis is a curable disease as long as early detection and rational drug use throughout the course of treatment.

Period effects usually reflect an impact of a given time that directly influence disease incidence and mortality on all age groups or birth cohorts, mainly generated by external macro factors like social, economic and medical levels. The decline of TB notification with period suggests the effectiveness of improving economic conditions to reduce tuberculosis progress rates, while the trend was not observed in TB mortality. The RR of period effect on TB mortality nearly kept stable from 2004 to 2013 and appeared to rise since 2013, probably induced by the abnormally high TB mortality data for 2018.. Over the past 15 years, the RR of period effect on TB notification and mortality barely changed, which reminded us that more attentionshould be paid in new detection methods and treatment techniques.

The role of age in variations of disease notification and mortality is often well understood, but the association of birth cohort with disease notification and mortality is difficult to understand. The cohort effect, also called generation effect, is considered to associate with some specific social events like wars, baby boom, great depression and so forth, reflecting different RR of disease incidence and mortality among different generations [35,36]. In this study, the cohort effect showed that the RR of TB notification and mortality decreased with the birth cohort, and the later people were born, the lower cohort effect risk got. The values of cohort effect on TB notification and mortality were less than 1.0 since 1978 when China began to reform and open, and even reached approximately 0.1 in the birth cohort of 2014-2018. Turbulent society, frequent wars, negative economy and productivity filled in early years of China, people were born in that time experienced higher risk of TB notification and mortality. The year of 1978, the beginning of reform and open policy, was a turning point for TB notification and mortality. People born in thattime experienced lower risk ofTB notification and mortality. There was a drop in TB notification (Fig.4a) among the cohort born in 1994. The government of China loaned from the World Bank to fully implement the strategy of Directly Observed Treatment Short-course (DOTS) in 13 Chinese provinces. Apparently, this made achievements. It also shows that tuberculosis is a disease closely associated to economy, society and health services. Compared with age and period effects, the cohort effects of TB notification and mortality are no longer obvious.

This study also has some limitations. For example, because of the data availability and model requirements, the data applied in the age-period-cohort model analysis only contained 15 years. It was not conducted at the gender, provinces, or urban and rural level, so we could not observe more detailed information that influences TB notification and mortality trends in China mainland. Besides, the published data of TB notification and mortality were only a fraction of actual TB cases and deaths in the population. The observed TB notification trend reflect the notified TB cases instead of all cases (known and unknown). Similarly, the observed TB mortality trend reflect patients who receive treatment instead of all patients. Thus, true incidence and mortality trends may be different from what has been observed in this study to a certain extent. In conclusion, hoping the analysis of TB notification and mortality trends between 2004 and 2019 and the estimation of the age, period and cohort effects will provide some evidence, references and insights for TB prevention and control in China mainland.

5. Conclusions

Tuberculosis notification and mortality maintained a low level at the age of 15. Young adults (15-34) and the elderly (50-84) are two risk groups of tuberculosis notification, especially for people who aged 20-24 and 70-74. Those aged 60 and over are risk people of tuberculosis mortality. The period effect helped to decrease tuberculosis notification instead of mortality. Later birth cohorts experienced lower TB notification and mortality than earlier birth cohorts, and the value of cohort effect risk was predicted to become much lower in the future. Moreover, the upward trend of tuberculosis mortality in China mainland residents in recent yearsdeserves our vigilance .

Author Contributions: Conceptualization and design of this study, W.W. and L.W.; funding acquisition, W.W.; data collection, W.W. and L.W.; statistical analysis and writing of manuscript, L.W.; revision of manuscript, W.W. All authors approved the published version of this manuscript.

Funding: This study was granted by the National Key Scientific and Technological Project against Major Infectious Diseases (Grant No. 2017ZX10201302-007-003), the Major Science and Technology Projects of Zhejiang Province (Grant No. 2014C03034), the National Natural Science Foundation of China (Grant No. 82073612).

Institutional Review Board Statement: Not applicable.

Informed Consent Statement: Not applicable.

Data Availability Statement: http://www.phsciencedata.cn

Acknowledgments: The authors thank Chinese Center for Disease Control and Prevention for the data.

Conflicts of Interest: The authors declare no conflict of interest.

References

1. Kolia-Diafouka, P.; Carrère-Kremer, S.; Lounnas, M.; Bourdin, A.; Kremer, L.; Van de Perre, P.; Godreuil, S.; Tuaillon, E. Detection of Mycobacterium tuberculosis in paucibacillary sputum: performances of the Xpert MTB/RIF ultra compared to the Xpert MTB/RIF, and IS6110 PCR. *Diagn Microbiol Infect Dis* **2019**, *94*, 365-370, doi:10.1016/j.diagmicrobio.2019.02.008.
2. Pasa, F.; Golkov, V.; Pfeiffer, F.; Cremers, D.; Pfeiffer, D. Efficient Deep Network Architectures for Fast Chest X-Ray Tuberculosis Screening and Visualization. *Sci Rep* **2019**, *9*, 6268, doi:10.1038/s41598-019-42557-4.
3. Yang, S.; Wu, J.; Ding, C.; Cui, Y.; Zhou, Y.; Li, Y.; Deng, M.; Wang, C.; Xu, K.; Ren, J.; et al. Epidemiological features of and changes in incidence of infectious diseases in China in the first decade after the SARS outbreak: an observational trend study. *Lancet Infect Dis* **2017**, *17*, 716-725, doi:10.1016/s1473-3099(17)30227-x.
4. Harding, E. WHO global progress report on tuberculosis elimination. *Lancet Respir Med* **2020**, *8*, 19, doi:10.1016/s2213-2600(19)30418-7.
5. Zuo, Z.; Wang, M.; Cui, H.; Wang, Y.; Wu, J.; Qi, J.; Pan, K.; Sui, D.; Liu, P.; Xu, A. Spatiotemporal characteristics and the epidemiology of tuberculosis in China from 2004 to 2017 by the nationwide surveillance system. *BMC Public Health* **2020**, *20*, 1284, doi:10.1186/s12889-020-09331-y.
6. Jiang, Y.; Dou, X.; Yan, C.; Wan, L.; Liu, H.; Li, M.; Wang, R.; Li, G.; Zhao, L.; Liu, Z.; et al. Epidemiological characteristics and trends of notifiable infectious diseases in China from 1986 to 2016. *J Glob Health* **2020**, *10*, 020803, doi:10.7189/jogh.10.020803.

7. Zhu, W.; Wang, Y.; Li, T.; Chen, W.; Wang, W. Gap to End-TB targets in eastern China: A joinpoint analysis from population-based notification data in Zhejiang Province, China, 2005-2018. *Int J Infect Dis* **2021**, *104*, 407-414, doi:10.1016/j.ijid.2021.01.007.
8. Meira, K.C.; Silva, G.; Dos Santos, J.; Guimarães, R.M.; de Souza, D.L.B.; Ribeiro, G.P.C.; Dantas, E.S.O.; Carvalho, J.B.L.; Jomar, R.T.; Simões, T.C. Analysis of the effects of the age-period-birth cohort on cervical cancer mortality in the Brazilian Northeast. *PLoS One* **2020**, *15*, e0226258, doi:10.1371/journal.pone.0226258.
9. Li, Z.; Wang, P.; Gao, G.; Xu, C.; Chen, X. Age-period-cohort analysis of infectious disease mortality in urban-rural China, 1990-2010. *Int J Equity Health* **2016**, *15*, 55, doi:10.1186/s12939-016-0343-7.
10. Ding, C.; Wang, S.; Shangguan, Y.; Feng, X.; Guo, W.; Shi, P.; Ji, Z.; Xu, K. Epidemic Trends of Tuberculosis in China from 1990 to 2017: Evidence from the Global Burden of Disease Study. *Infect Drug Resist* **2020**, *13*, 1663-1672, doi:10.2147/IDR.S249698.
11. Wen, H.; Xie, C.; Wang, L.; Wang, F.; Wang, Y.; Liu, X.; Yu, C. Difference in Long-Term Trends in COPD Mortality between China and the U.S., 1992 ⁻ 2017: An Age ⁻ Period ⁻ Cohort Analysis. *Int J Environ Res Public Health* **2019**, *16*, doi:10.3390/ijerph16091529.
12. Iqbal, S.A.; Winston, C.A.; Bardenheier, B.H.; Armstrong, L.R.; Navin, T.R. Age-Period-Cohort Analyses of Tuberculosis Incidence Rates by Nativity, United States, 1996-2016. *Am J Public Health* **2018**, *108*, S315-S320, doi:10.2105/AJPH.2018.304687.
13. Wu, P.; Cowling, B.J.; Schooling, C.M.; Wong, I.O.; Johnston, J.M.; Leung, C.C.; Tam, C.M.; Leung, G.M. Age-period-cohort analysis of tuberculosis notifications in Hong Kong from 1961 to 2005. *Thorax* **2008**, *63*, 312-316, doi:10.1136/thx.2007.082354.
14. Lee, L.T.; Chen, C.J.; Lee, W.C.; Luh, K.T.; Hsieh, W.C.; Lin, R.S. Age-period-cohort analysis of pulmonary tuberculosis mortality in Taiwan: 1961 to 1990. *J Formos Med Assoc* **1994**, *93*, 657-662.
15. Yun, J.W.; Son, M. Forecasting Cause-Specific Mortality in Korea up to Year 2032. *J Korean Med Sci* **2016**, *31*, 1181-1189, doi:10.3346/jkms.2016.31.8.1181.
16. Cui, Y.; Shen, H.; Wang, F.; Wen, H.; Zeng, Z.; Wang, Y.; Yu, C. A Long-Term Trend Study of Tuberculosis Incidence in China, India and United States 1992-2017: A Joinpoint and Age-Period-Cohort Analysis. *Int J Environ Res Public Health* **2020**, *17*, doi:10.3390/ijerph17093334.
17. Llanes-Álvarez, C.; Llano, J.M.A.; Álvarez-Navares, A.I.; Roncero, C.; Pastor-Hidalgo, M.T.; Garmendia-Leiza, J.R.; Andrés-Alberola, I.; Franco-Martín, M.A. Hospitalization and Socio-Health Care for Dementia in Spain. *J Clin Med* **2020**, *9*, doi:10.3390/jcm9123875.
18. KimHJ , F., FeuerEJ, et al. Permutation tests for joinpoint regression with applications to cancer rates. *Stat Med* **2000**, *19*, 335-351.
19. Goovaerts, P.; Xiao, H. Geographical, temporal and racial disparities in late-stage prostate cancer incidence across Florida: a multiscale joinpoint regression analysis. *Int J Health Geogr* **2011**, *10*, 63, doi:10.1186/1476-072x-10-63.
20. Hagiya, H.; Koyama, T.; Zamami, Y.; Minato, Y.; Tatebe, Y.; Mikami, N.; Teratani, Y.; Ohshima, A.; Shinomiya, K.; Kitamura, Y.; et al. Trends in incidence and mortality of tuberculosis in Japan: a population-based study, 1997-2016. *Epidemiol Infect* **2018**, 1-10, doi:10.1017/S095026881800290X.
21. Mason, K.O.; W. M. Mason; H.H. Winsborough; W.K. Poole. Some methodological issues in cohort analysis of archival data. *Am Sociol Rev* **1973**, *38*, 242-58.
22. Yang Y, Schulhofer-Wohl S, Fu WJ, Land KC. The intrinsic estimator for age-period-cohort analysis: what it is and how to use it. *Am J Sociol* **2008**, *113*, 1697–736.
23. Luo, L. Assessing validity and application scope of the intrinsic estimator approach to the age-period-cohort problem. *Demography* **2013**, *50*, 1945-1967, doi:10.1007/s13524-013-0243-z.
24. Li, J.; Li, B.; Zhang, F.; Sun, Y. Urban and rural stroke mortality rates in China between 1988 and 2013: An age-period-cohort analysis. *J Int Med Res* **2017**, *45*, 680-690, doi:10.1177/0300060516664241.
25. Liu, X.; Yu, C.; Bi, Y.; Zhang, Z.J. Trends and age-period-cohort effect on incidence and mortality of prostate cancer from 1990 to 2017 in China. *Public Health* **2019**, *172*, 70-80, doi:10.1016/j.puhe.2019.04.016.
26. Wang, L.; Yu, C.; Liu, Y.; Wang, J.; Li, C.; Wang, Q.; Wang, P.; Wu, S.; Zhang, Z.J. Lung Cancer Mortality Trends in China from 1988 to 2013: New Challenges and Opportunities for the Government. *Int J Environ Res Public Health* **2016**, *13*, doi:10.3390/ijerph13111052.
27. Tzeng, I.S.; Chen, K.H.; Lee, Y.L.; Yang, W.S. Trends and Age-Period-Cohort Effects of Fertility Rate: Analysis of 26,224 Married Women in Taiwan. *Int J Environ Res Public Health* **2019**, *16*, doi:10.3390/ijerph16244952.
28. Li, T.; Du, X.; Chen, W.; Huang, F.; Zhao Y.L.; Zhang H. Review and prospect of information monitoring and control of tuberculosis management in China (in Chinese). *Chin J Antituberc* **2020**, *42*, 657-661.
29. Schwartz, N.G.; Price, S.F.; Pratt, R.H.; Langer, A.J. Tuberculosis - United States, 2019. *MMWR Morb Mortal Wkly Rep* **2020**, *69*, 286-289, doi:10.15585/mmwr.mm6911a3.
30. Kirenga, B.J.; Ssengooba, W.; Muwonge, C.; Nakiyingi, L.; Kyaligonza, S.; Kasozi, S.; Mugabe, F.; Boeree, M.; Joloba, M.; Okwera, A. Tuberculosis risk factors among tuberculosis patients in Kampala, Uganda: implications for tuberculosis control. *BMC Public Health* **2015**, *15*, 13, doi:10.1186/s12889-015-1376-3.
31. Narita, M.; Sullivan Meissner, J.; Burzynski, J. Use of Modeling to Inform Tuberculosis Elimination Strategies. *Am J Respir Crit Care Med* **2020**, *201*, 272-274, doi:10.1164/rccm.201910-2061ED.
32. ICN(International Council of Nurses). Tuberculosis guidelines.
33. Wang, F.; Mubarik, S.; Zhang, Y.; Wang, L.; Wang, Y.; Yu, C.; Li, H. Long-Term Trends of Liver Cancer Incidence and Mortality in China 1990-2017: A Joinpoint and Age-Period-Cohort Analysis. *Int J Environ Res Public Health* **2019**, *16*, doi:10.3390/ijerph16162878.

34. Negin, J.; Abimbola, S.; Marais, B.J. Tuberculosis among older adults--time to take notice. *Int J Infect Dis* **2015**, *32*, 135-137, doi:10.1016/j.ijid.2014.11.018.
35. Fletcher, J.M. The effects of in utero exposure to the 1918 influenza pandemic on family formation. *Econ Hum Biol* **2018**, *30*, 59-68, doi:10.1016/j.ehb.2018.06.004.
36. Robinson, W.R.; Utz, R.L.; Keyes, K.M.; Martin, C.L.; Yang, Y. Birth cohort effects on abdominal obesity in the United States: the Silent Generation, Baby Boomers and Generation X. *Int J Obes (Lond)* **2013**, *37*, 1129-1134, doi:10.1038/ijo.2012.198.
